# Self-reported physical activity in people with limb-girdle muscular dystrophy and Charcot-Marie-Tooth disease in Norway

**DOI:** 10.1186/s12891-020-03246-w

**Published:** 2020-04-13

**Authors:** Aristomo Andries, Marleen R. van Walsem, Jan C. Frich

**Affiliations:** 1 Institute of Health and Society, University of Oslo, P.O. Box 1089, N-0318 Oslo, Blindern Norway; 2 Department of Neurohabilitation, Oslo University Hospital, P.O. Box 4956, N-0424 Oslo, Nydalen Norway; 3 Research Centre for Habilitation and Rehabilitation Models and Services, Institute of Health and Society, University of Oslo, P.O. Box 1089, N-0318 Oslo, Blindern Norway

**Keywords:** Neuromuscular disease, Physical activity, Limb-girdle muscular dystrophy, Charcot-Marie-Tooth disease, Hereditary motor and sensory neuropathy, Sedentary, Disability, Habilitation

## Abstract

**Background:**

Physical activity is associated with positive health effects, but individuals with neuromuscular disease (NMD) may experience constraints being physically active. There is a gap in the literature on the activity level of people with NMDs, and therefore we did a study to determine the physical activity level in people with Limb-Girdle muscular dystrophy (LGMD) and Charcot-Marie-Tooth disease (CMT).

**Methods:**

This study used a cross-sectional design to obtain self-reported physical activity and sitting time among individuals with LGMD and CMT who were recruited from the Norwegian registry for hereditary and congenital neuromuscular diseases.

**Results:**

A total of 127 respondents who filled out questionnaires about either physical activity or sitting time were included in the analysis. Seventy (55.1%) had a diagnosis of CMT and 57 (44.9%) had a diagnosis of LGMD. Seventy-three (57.5%) respondents were female and 54 (42.5%) were male. Among the 108 respondents with available physical activity data, 44.4% reported being physically inactive. Among the 109 respondents with available sitting time data, the average sitting time was 8.6 h. Longer sitting time was associated with higher physical inactivity.

**Conclusion:**

Among people with LGMD and CMT in our study, 55.6% reported being physically active. Respondents with LGMD and CMT reported longer sitting time and less physical activity compared with healthy respondents in other studies. Further research should explore variables and measures that can promote physical activity among people with neuromuscular conditions.

## Background

Physical activity is important for health and impacts personal health and well-being [1–3]. World Health Organization (WHO) has recommended at least 150 min of moderate-intensity physical activities or 75 min of vigorous-intensity activities per week [2]. Daily physical activities are not limited to leisure time physical activities such as non-occupational related exercise at fitness centre, tennis court, or jogging track, but also physical activities at work [4]. The amount of energy used during physical activities is calculated in kilocalories (kcal) [4, 5]. A metabolic equivalent (MET) denotes the amount of oxygen consumption at rest [6] and allows for measuring and comparing the intensity between various physical activities [6]. In studies that have mapped individuals’ self-reported physical activity using International Physical Activity Questionnaire (IPAQ), 150 min of moderate-intensity activity during a week corresponds to a minimum of 600 MET-minutes/week [7, 8].

Neuromuscular disease (NMD) may limit a person’s ability for being physically active [9–11]. In Norway, Charcot-Marie-Tooth disease (CMT) and Limb-girdle muscular dystrophy (LGMD) are among the most prevalent NMDs, representing 19 and 15% of patients registered in the Norwegian registry for hereditary and congenital neuromuscular diseases [12]. Both CMT and LGMD affect the patient’s extremities and may cause ambulatory problems [9, 10]. The systemic involvement of LGMD in the cardiovascular system and CMT in the diaphragm respiratory muscles may further limit physical functioning of affected individuals [13–15]. Although regular physical activities for individuals with LGMD are beneficial, over-exercising or high-resistance strength training may need to be avoided [16, 17].

Globally, Bauman et al. [7] reported that the proportions of physically active individuals in 20 countries varied from 56.6 to 93.1%. Hallal et al. [18] conducted analysis on the physical activity data from WHO data repository in 2011 and found that 31.1% adults in 122 countries did not meet recommended physical activity levels. A recent study also found a similar physical activity level [19]. These international studies used self-reported physical activity data from the IPAQ. In a nationwide survey, the Norwegian Directorate of Health reported that 67% of the survey participants in self-reported physical activity data (collected using the IPAQ) met the national physical activity recommendation. However, in the accelerometer data, only one-third of the survey participants representing the general population met the country’s physical activity recommendation [8].

Due to the neuromuscular and skeletal issues experienced by people with LGMD and CMT, the challenge to be physically active could be even greater [15]. It is important to involve people with NMD in activities that promote a healthy lifestyle, such as being physically active [20]. It is also important that people with disabilities are included in research that can contribute to promoting their health and well-being [21, 22]. There is a gap in the scientific literature on the activity level of people with NMDs, and therefore we did a study to map the level of physical activity among people with LGMD and CMT in Norway.

## Methods

### Procedures

We did an observational study with a cross-sectional design. We chose individuals with LGMD and CMT because these groups are likely to experience disabilities, and they represented a large proportion of patients in the Norwegian registry for hereditary and congenital neuromuscular diseases (University Hospital of North Norway). We sent a mail invitation about the study to individuals with LGMD and CMT who were registered, and who had consented to participate in research. The mail included a set of paper-based questionnaires with an option to use an online-based questionnaire. People who consented to participate in the study replied either using a return envelope or through the online survey. We sent one reminder. The survey was open between September and December 2017.

### Participants

The inclusion criteria for this study were adults aged 18–65 years old per July 1st, 2017 who had a diagnosis of LGMD or CMT, registered in the Norwegian registry for hereditary and congenital neuromuscular diseases. We excluded individuals that were unable to walk (e.g. bed ridden or hospitalized in bed) and those who had major surgery within 3 months prior the study invitation. We used a sample size calculator from Centre for Biomathematics at Columbia University Medical Centre website (http://www.biomath.info/power/index.html). We used a total MET-minutes/week output from another study using the IPAQ in two different groups: people with fibromyalgia (the mean value of total MET-minutes/week was 2741, SD ± 3081) and a control group (4338, SD ± 3232) [23]. Assuming a mean difference of physical activity level in the two diseases groups and an equal proportion of participants with both conditions, we calculated the sample size with a *p*-value of 0.05 and power of 80%. Based on these assumptions, we calculated that the total minimum of respondents for both conditions was 126. We invited almost twice as many patients to accommodate a low response rate. From 306 potential respondents in the registry, 250 individuals were randomly selected using the randomisation function in Microsoft Excel.

### Data and measurements

**Fig. 1:**
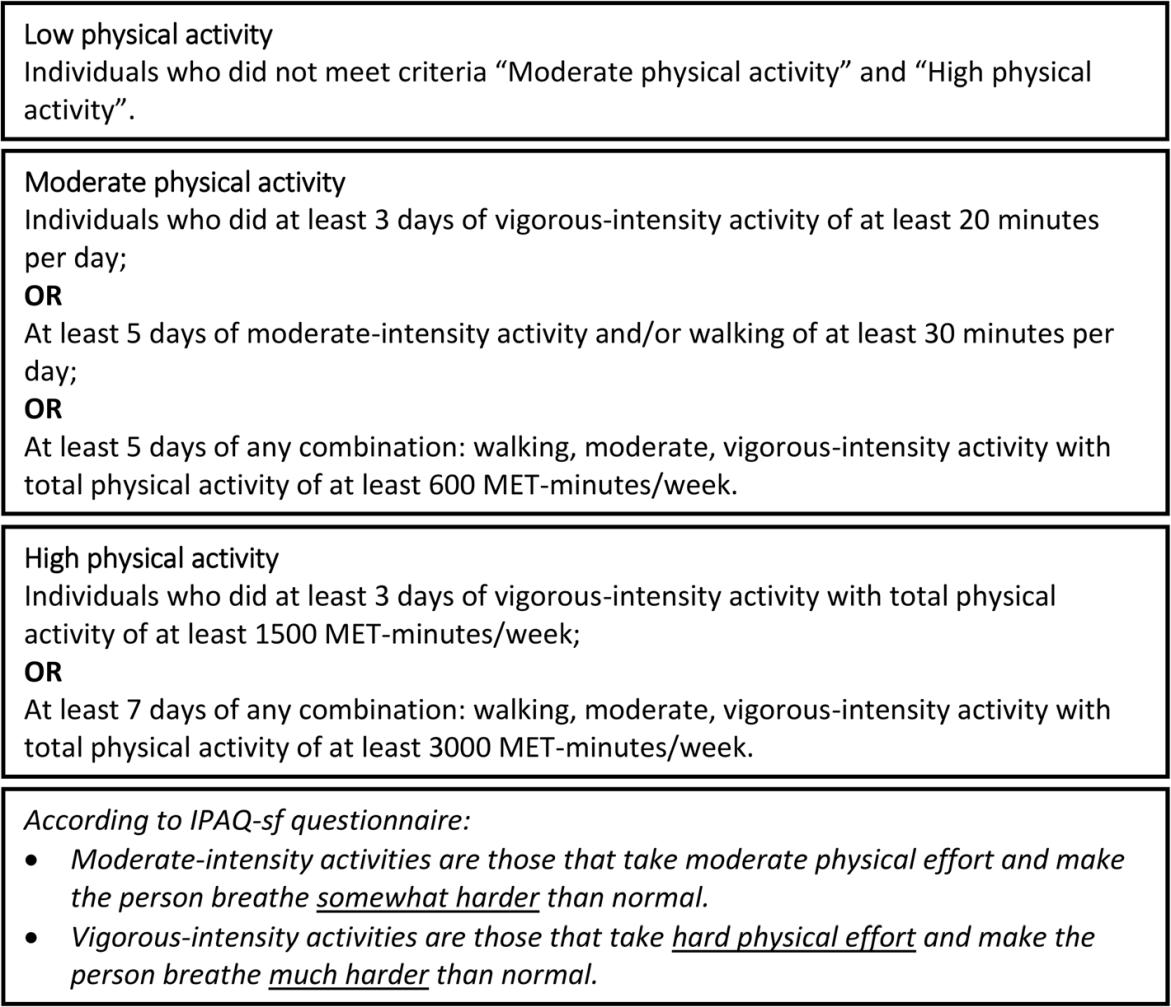
Summary of IPAQ-sf physical activity categories [25]

Physical activity level in this study was self-reported. Each participant completed the International Physical Activity Questionnaire short form (IPAQ-sf). The questionnaire records physical activities for the last 7 days in all domains, including work-related and leisure time physical activities [24]. The IPAQ-sf was used in a previous survey among the general adult Norwegian population [8] and has been used in many other studies involving adults aged 18–65 years old [7, 8]. The instrument has inter-class correlations of 0.7–0.8 for retest reliability and validity ρ = 0.3 for comparison with the accelerometer [7, 24]. Results from IPAQ-sf are presented as the value of MET-minutes/week for continuous variables and as physical activity categories for categorical variables. We used the IPAQ-sf scoring protocol version 2005 to calculate the results [25]. The categorical variable for physical activity included three different categories (Fig. 1). The “moderate” and “high” physical activity categories were combined into the “physically active” group. This group represents respondents who met the minimum recommended physical activity level [1–3, 8]. The “low” physical activity category represents the “physically inactive” group. Variables sex, age, diagnosis, and place of living were obtained from the neuromuscular register. Other sociodemographic variables were obtained from the questionnaire.

We divided respondents in four age groups: a) ≤ 20, b) 21–40, c) 41–60, and d) > 60 years. The data on sitting time was obtained from the IPAQ-sf [24]. We divided sitting time according to the quartiles into four groups: a) < 5 h, b) 5 to less than 8 h, c) 8 to less than 11 h, and d) ≥ 11 h. We grouped the reported sitting time in groups in accordance with previous studies [26, 27]. The grouping reflects the common practice in the community, such as the requirement to sit in the office for 8 h or more. To our knowledge, there is no well-established cut-off value where sitting time increases risk of diseases [26, 28].

### Statistical analyses

For continuous variables with a normal distribution, means and standard deviations (SD) were calculated. The median and interquartile range (IRQ) were presented for variables that were not normally distributed. Categorical variables were reported using calculations of proportions. We assessed the relationships between variables of participants’ characteristics and physical activity level as a dependent outcome variable using T-Test or Mann-Whitney U test, depending on the distribution of the data. Chi-square or Fischer’s exact tests were used to analyse relationships between categorical variables. We further compared the relationships of study variables with physical activity using logistic regression analysis [29]. Statistically significant variables in the univariate analysis were included in the multiple logistic regression. Variables age groups, sex, and diagnosis were also included in the multiple logistic regression to control for heterogeneity of the subjects. The results of logistic regression analyses were presented as odds ratio (OR) with 95% confidence interval (CI). Data from non-responders and participants excluded from physical activity or sitting time analysis were analysed to identify biases. The level of significance was set at 0.05. We used SPSS statistical software version 25 manufactured by International Business Machines (IBM) Corporation to process the study data.

## Results

**Fig. 2:**
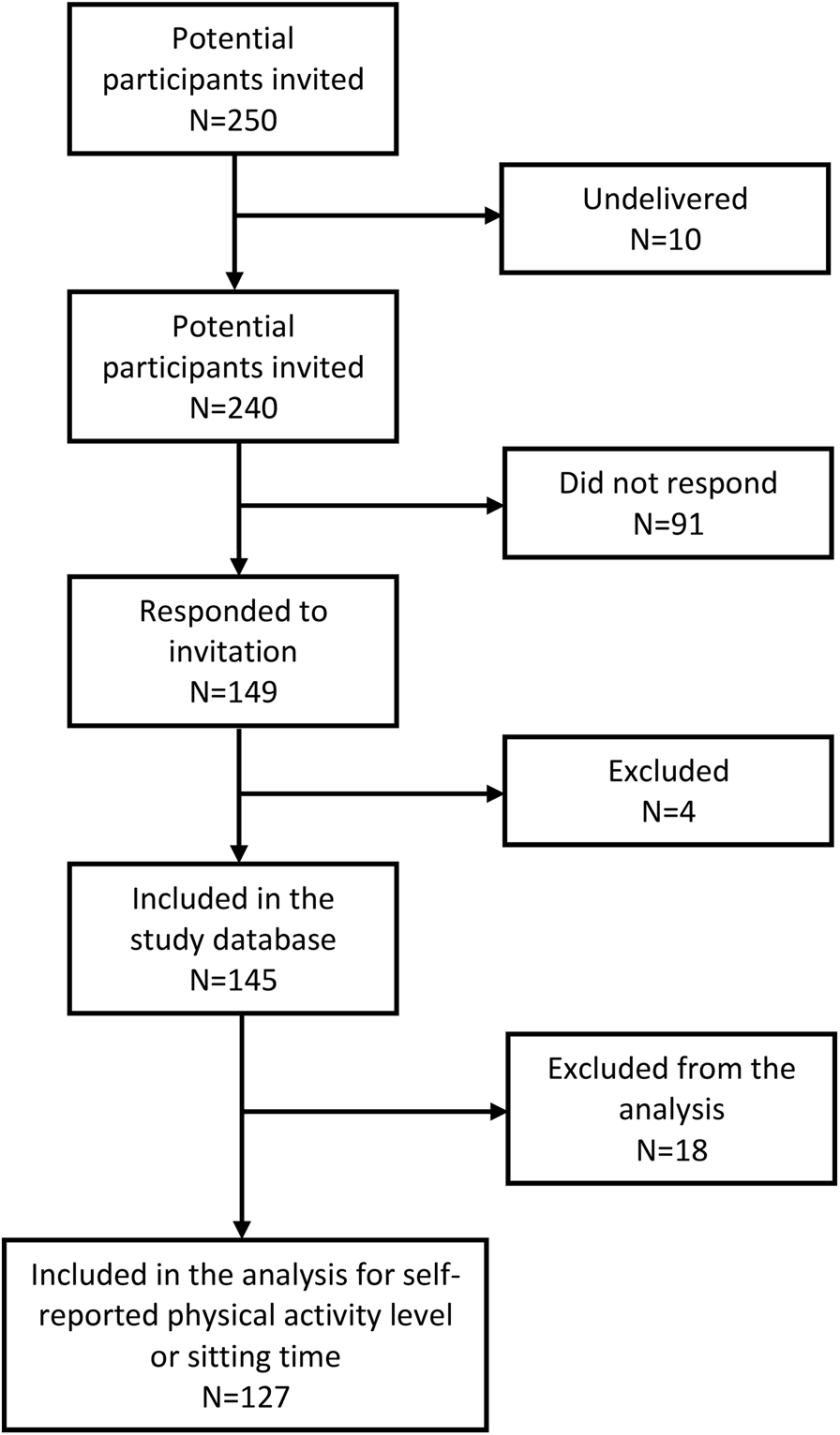
Flowchart of study participation

A total of 149 individuals responded to our study (response rate of 62.1%). From 149 responders, we excluded four respondents because they reported having a diagnosis other than LGMD or CMT. Of the 145 respondents with LGMD or CMT diagnosis, we included 127 respondents who met the criteria for analysis of self-reported physical activity level based on the IPAQ-scoring protocol or sitting time [25]. The remaining 18 respondents opted to answer “forget” or “do not know” in physical activity components and sitting time of the IPAQ-sf questionnaire. Consequently, these 18 respondents were not included in the analysis. Figure 2 illustrates the participant inclusion flow as described above.

### Participant characteristics

Among the 127 study participants, 70 (55.1%) had a diagnosis of CMT and 57 (44.9%) were diagnosed with LGMD. Seventy-three (57.5%) of the respondents were female. Table 1 shows the proportion of the variables age, sex, diagnosis, and other sociodemographic variables of the study respondents.

**Table 1:** Characteristics of the 127 respondents with LGMD and CMT included in the analysis

Variables	***n*** (%)
**Age group (in years)**	
≤ 20	6 (4.7)
21–40	38 (30.0)
41–60	61 (48.0)
> 60	22 (17.3)
**Sex**	
Female	73 (57.5)
Male	54 (42.5)
**Place of living**	
< 10,000 population	56 (44.1)
≥ 10,000 population	71 (55.9)
**Civil status**	
Married	84 (66.1)
Not married	43 (33.9)
**Education**	
Primary to upper secondary school	65 (51.2)
College or more than 13 years education	62 (48.8)
**Employment**	
Yes	102 (80.3)
No/retired	25 (19.7)
**Diagnosis**	
Charcot-Marie-Tooth disease	70 (55.1)
Limb-girdle muscular dystrophy	57 (44.9)

### Self-reported level of physical activity (MET and categorical) in LGMD and CMT

A total of 108 individuals were included in the descriptive analysis of self-reported physical activity level. The results for level of physical activity were skewed to the right (skewness statistic was 2.036 with standard error (SE) 0.233). The median of self-reported physical activity level was 1194.00 (IQR = 2651.50). One respondent who scored 13,518.00 MET-minutes/week was identified as an outlier. However, there was no error in the data of this particular case and was therefore kept in the analyses. Table 2 shows the level of self-reported physical activity (in MET-minutes/week) for each of the intensity activities used in the IPAQ-sf [25].

**Table 2:** The self-reported physical activity level (in MET-minutes/week) for walking, moderate, and vigorous-intensity activities (*n* = 108)

Self-reported physical activity component	Median (IQR)
Walking	198.00 (792.00)
Moderate-intensity	240.00 (960.00)
Vigorous-intensity	0.00 (1050.00)

The IPAQ-sf data were grouped into physical activity category. Forty-eight (44.4%) respondents were in the “low” physical activity group, 30 (27.8%) were in the “moderate” group and 30 (27.8%) were in the “high” group. Since we considered people with moderate and high physical activity to meet the minimum recommendation of being physically active [1–3, 8], we combined these groups into one group of “physically active” individuals. Consequently, 48 (44.4%) respondents were categorised as “physically inactive” and 60 (55.6%) respondents were “physically active”.

Table 3 shows the self-reported physical activity as the level in MET-minutes/week and as a category (inactive vs. active) for the two different diagnosis groups. For the group of respondents with CMT, the median physical activity level was 1394.25 (IQR = 3078.25). In the LGMD diagnosis group, the median of physical activity level was 947.00 (IQR = 2659.50). There was no significant difference in self-reported physical activity between the two groups of diagnosis.

**Table 3:** Physical activity category and level in 108 individuals with CMT and LGMD

Diagnosis groups	Physical activity category^**a**^		Physical activity level^**b,c**^
	Inactive (%)	Active (%)	Median (IQR)
CMT (*n* = 58)	22 (37.9)	36 (62.1)	1394.25 (3078.25)
LGMD (*n* = 50)	26 (52.0)	24 (48.0)	947.00 (2659.50)
**Both groups combined**	**48 (44.4)**	**60 (55.6)**	**1194.00 (2651.50)**

*CMT* Charcot-Marie-Tooth disease, *LGMD* Limb-girdle muscular dystrophy.

^a^*p* = 0.142 using Pearson’s Chi-square test for physical activity category (significant at *p* < 0.05)

^b^*p* = 0.053 using Mann-Whitney U test for physical activity level difference in both diagnoses (significant at *p* < 0.05)

^c^Physical activity level in MET-minutes/week

### Sitting time

The IPAQ-sf instrument provides information about participants’ sitting time. A total of 109 respondents were analysed for sitting time. Sitting time was normally distributed (Kolmogorov-Smirnov (*p* = 0.200) and Shapiro-Wilk (*p* = 0.307)) [29]. Table 4 presents the sitting time (in hours) for the two different diagnosis groups. We found no difference in sitting time between the diagnoses.

**Table 4:** Sitting time (in hour) based on diagnosis groups (*n* = 109)

Diagnosis groups	Mean (±SD)
CMT (*n* = 59)	8.3 (3.7)^a^
LGMD (*n* = 50)	9.1 (4.0)^a^
**Both groups combined**	**8.6 (3.9)**

*CMT* Charcot-Marie-Tooth disease, *LGMD* Limb-girdle muscular dystrophy.

^a^*p* = 0.282 using independent sample T-test (significant at *p* < 0.05)

**Fig. 3:**
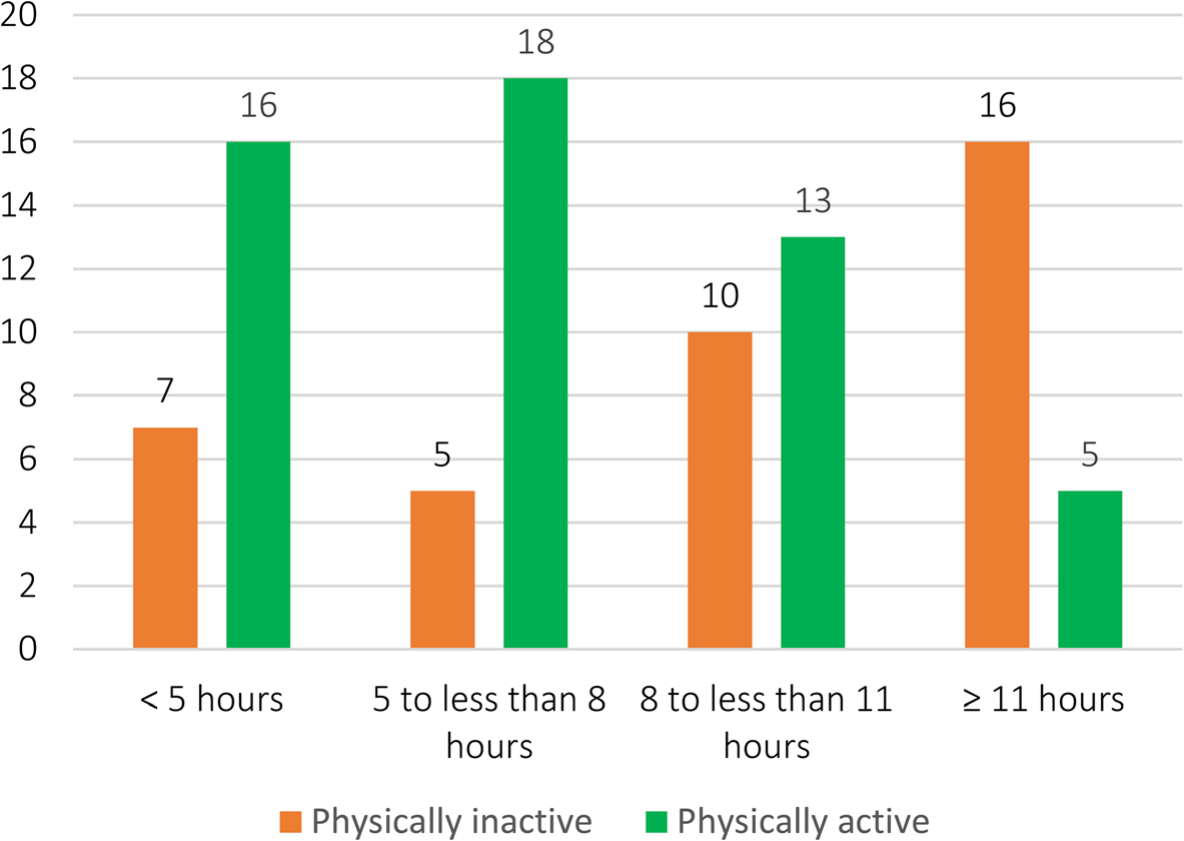
Physical activity categories for each sitting time groups (*n* = 90)

From 127 respondents, 90 have data for both physical activity and sitting time. These data were used to explore the relationship between sitting time and physical activity level. Figure 3 shows physical activity categories (physically active and physically inactive) based on the sitting time groups.

### The relationships between sample characteristics and sitting time, and self-reported physical activity

The associations between age, sex, diagnosis, sociodemographic variables, and sitting time with the categorical variable for self-reported physical activity (“physically inactive” and “physically active”) as the dependent variable is presented in Table 5. The predicted probability for these associations was “physically inactive”. In both univariate and multivariable logistic regression, there were associations between sitting time and physical activity. Sitting time group ≥11 h was the reference value for the other sitting time groups and represented the most sedentary condition. Considering variables age, sex, and diagnosis in the logistic regression analysis, the odds of being physically inactive was reduced by 76% (95% CI 5–94%, *p* = 0.042) in the group of respondents with sitting time 8 h to < 11 h compared to the reference group with sitting time ≥ 11 h. In the sitting time 5 h to < 8 h group, the odds reduction was 92% (95% CI 65–98%, *p* = 0.001) in comparison with the reference group. For the sitting time group < 5 h, the odds of being physically inactive was 86% reduced (95% CI 46–97%, *p* = 0.005).

**Table 5:** Associations between sex, age groups, diagnosis, and sitting time with dependent variable physical activity with being “physically inactive” as predicted probability in univariate and multivariable logistic regression analysis (*n* = 90)

Variables	Univariate		Multivariable	
	OR^**a**^ (95% CI)	***p*** value	OR^**a**^ (95% CI)	***p*** value
**Age groups (in years)**				
≤ 20	1		1	
21–40	1.12 (0.09–14.20)	0.927	2.13 (0.10–46.62)	0.630
41–60	1.62 (0.14–19.07)	0.703	3.26 (0.17–63.41)	0.435
> 60	1.75 (0.13–23.70)	0.674	2.54 (0.12–56.06)	0.555
**Sex**				
Female	1		1	
Male	0.81 (0.35–1.87)	0.622	0.67 (0.24–1.87)	0.442
**Place of living**				
< 10,000 population	1		–	–
≥ 10,000 population	0.71 (0.30–1.67)	0.434		
**Civil status**				
Married	1		–	–
Not married	0.84 (0.34–2.08)	0.705		
**Education**				
Primary to upper secondary school	1		–	–
College or > 13 years education	1.92 (0.81–4.56)	0.137		
**Employment**				
Yes	1		–	–
Retired	2.04 (0.64–6.48)	0.225		
**Diagnosis**				
CMT	1		1	
LGMD	2.03 (0.87–4.75)	0.102	2.03 (0.78–5.30)	0.147
**Sitting time (in hour)**				
≥ 11 h	1		1	
8 to less than 11 h	0.24 (0.07–0.88)	0.032*	0.24 (0.06–0.95)	0.042*
5 to less than 8 h	0.09 (0.02–0.36)	0.001*	0.08 (0.02–0.35)	0.001*
< 5 h	0.14 (0.04–0.52)	0.004*	0.14 (0.03–0.54)	0.005*

*CMT* Charcot-Marie-Tooth disease, *LGMD* Limb-girdle muscular dystrophy.

^a^ Odds ratio (OR) predicted probability is of membership for physically inactive

* Significant at *p* < 0.05

### Analyses of characteristics of non-responders

There is a significant age difference between those who initially responded to the study invitation and those who did not. The study responses came from an older age group. When we analysed these responses, we further excluded 18 respondents. There was no age difference between the respondents who were not included in the physical activity or sitting time analysis and the 127 respondents who were included.

## Discussion

The study showed a median of self-reported physical activity level of 1194 MET-minutes/week. The physical activity level data were skewed to the right, therefore more than half of the respondents scored lower than the average. In comparison with the current recommendation of 150 min of moderate-intensity activity during a week (600 MET-minutes/week), the study result showed that the goal was achievable for more than half of the respondents [2, 3]. Accordingly, we found that 55.6% of study participants were in the physically active category.

In comparison with the global figures of self-reported physical activity, our study found a lower proportion of people in the physically active category. As reported in the study of Bauman et al. [7], the lowest proportion of physically active individual in a country was 56.6%. Considering only the subjective measurement with the same tool, the latest survey in Norway presented that 67% of the general adult population was physically active. However, the objective measurement from the same survey reported that only 32% were physically active [8]. Our study also found a higher proportion in the physically inactive group (44.4%) in comparison with international numbers in the studies by Hallal et al. [18] and Guthold et al. [19].

More than a quarter (27.8%) of our study participants reported an activity level above the minimal recommended level, as they were able to achieve a high level of physical activity. This could mean that even though both LGMD and CMT might affect them from early life, most of our study participants were physically active. Both patients with LGMD and CMT experience gradual decline in their physical function [9, 10]. Therefore, many of them are capable of being physically active in their adulthood. This finding also indicates that many of our respondents were able to be physically active beyond the minimum recommendation of physical activity set by the Norwegian Directorate of Health: at least 150 min of moderate-intensity activities per week [3]. For most people with LGMD and CMT, the existing public health campaign to promote physical activity seems to work. However, there is a need to explore measures to improve physical activity for the remaining people with LGMD and CMT with low physical activity in this study.

The present study did not find significant differences between respondents with LGMD and those with CMT in their ability to meet the minimum physical activity recommendation and the median of their physical activity level. These findings seem plausible since both LGMD and CMT share common characteristics, such as both affecting the patients’ extremities without causing paralysis [9, 10]. Good physical ability could also indicate that interventions to overcome their disease conditions have been put in place: as it is known that moderate-intensity exercise is beneficial for LGMD [16]. Further research is needed to explore the benefit of physical activity in people with various NMDs [30–32].

This study reported an average daily sitting time of 8.6 h for both respondents in LGMD and CMT groups combined. The amount of time spent sedentary was higher than in the general population reported in the study of Bauman et al. [26]. It was also higher than the average self-reported daily sitting time in the general population in Norway, which was 7.3 h for men and 6.9 h for women [8]. A study in Finland reported an average sitting time of 6.7 h/day for people with cardiovascular diseases [33]. Sedentary behaviour has been associated with increased risk of non-communicable diseases (NCDs) [26]. For example, a Norwegian study reported that sitting time ≥ 8 h/day was associated with 17% increased risk of diabetes incidence [34]. With longer sitting time duration, our study participants could be more at risk for NCDs in comparison with the average population in Norway and globally. Similar findings may also be true for other people with disability conditions [35].

Our study found associations between sitting time groups and physical activity. The groups with shorter sitting time duration were less likely to being physically inactive. We also identified that there were respondents who were still physically active in the group with longest sitting time, which suggested that individuals who were active can at the same time have much sedentary time [36]. On the other hand, it may also imply that the measurement tool was not sufficiently sensitive to capture active sitting condition, such as some exercises in sitting position [37, 38]. Further research could aim to explore more about active sitting condition in this group.

This study did not find significant associations between sex and age with the physical activity in people with LGMD and CMT. It is known that both CMT and LGMD have similar characteristics in both men and women [9, 10]. However, no significant difference of physical activity in age groups was counterintuitive with the general picture that younger individuals are potentially more physically active [39]. This may be explained as the result of response bias that many of our respondents came from older age group.

Few other studies have explored physical activity among people with disabilities due to NMDs [40–42]. In one of the studies, Saebu and Sørensen [40] obtained self-reported physical activity levels using the same instrument (IPAQ-sf) in the people with various disability conditions. They reported the average physical activity level was 1595 (SD ± 1985) MET-minutes/week, which was lower than the current study average (1901 MET-minutes/week) [40]. However, in the study by Saebu and Sørensen [40], only 14.7% of the participants were having disabilities due to muscle problem without further description about specific diagnoses. Other studies in people with CMT by Ramdharry et al. [41], and Anens et al. [42] used different instruments to collect information about physical activity. Ramdharry used objective measurement [41] and Anens used Physical Activity Disability Survey-Revised (PADS-R) questionnaire [42]. In Anens et al. [42] study, there was no description about physical activity level in METs. Because of differences in presenting the study data, it is difficult to compare our study results with these other studies.

### Strengths and limitations

We recruited study participants from a random sample from the Norwegian registry for hereditary and congenital neuromuscular diseases. This randomisation was an effort to minimise selection bias among the potential respondents [43]. We used a validated tool to measure physical activity and sitting time, which can be compared with the previous surveys national and internationally [7, 8, 19, 26, 40]. The IPAQ-sf also measures physical activities from all domains [24], which is a useful information to compare it with existing physical activity recommendations [2, 3]. The use of standardised tool could contribute as a baseline data for further follow up.

Of all NMD diagnoses, this study’s participants belong to only two disease groups. Therefore, the finding of this study cannot be generalised to other type of NMDs. Respondents were recruited from the Norwegian registry for hereditary and congenital neuromuscular diseases, and the study might not cover patients with NMD who have not been registered [12]. Although more than half (62.1%) responded to the study invitation, which was better than other physical activity surveys in people with disability [40, 42], not all responses were used in the analysis due to incomplete data and/or respondents being unable to recall their physical activity. This limitation is not uncommon for cross-sectional studies using questionnaires [43].

The use of IPAQ short version also did not give information about specific physical activity domains that the study participants performed [44]. Furthermore, there were discrepancies between subjective and objective physical activity measurements [8]. In this case, the subjective measurement tends to over report the level of physical activity [45]. In the absence of a gold standard for habitual physical activity measurement [46], we recommend the combination of a subjective tool such as the IPAQ and an objective instrument such as the accelerometer for follow up studies. The subjective tool can be used to obtain information about physical activity duration at a certain intensity and activity patterns. The objective tool can be used to confirm the duration of being active. Our study findings, showing that more than half of the study participants met the minimum physical activity recommendation, could be an overstatement.

## Conclusion

Among people with LGMD and CMT in our study, 55.6% reported being physically active. There is a potential for increased physical activity in these patients, and the need to develop initiative to promote physical activity in people with NMD. Further research should explore variables and measures that can promote physical activity among people with neuromuscular conditions.

## Data Availability

The datasets used and/or analysed during the current study are available from the corresponding author on reasonable request.

## Abbreviations

CI: Confidence interval
CMT: Charcot-Marie-Tooth disease
IBM: International Business Machines
IPAQ: International Physical Activity Questionnaire
IPAQ-sf: International Physical Activity Questionnaire short form
IRQ: Interquartile range
kcal: Kilocalories
LGMD: Limb-girdle muscular dystrophy
MET: Metabolic equivalent
NMD: Neuromuscular disease
OR: Odds ratio
PADS-R: Physical Activity Disability Survey-Revised
SD: Standard deviation
SE: Standard error
SPSS: Statistical Package for Social Sciences
TSD: *Tjenester for Sensitive* Data
WHO: World Health Organization
